# Progress in Medicine

**Published:** 1900-02-24

**Authors:** 


### Progress in Medicine.

### DISEASES OF THE NERVOUS SYSTEM.

**Relation of the Motor Nerves to the Tissues of the Body.**—In his presidential address to the Physiological Section of the British Association for the Advancement of Science, Langley[1] discussed this most interesting question. He said that in any broad classification of the body tissues, not more than two tissues are known to be supplied with approximate completeness with efferent nerve fibres, viz., the striated and unstriated muscular tissues. With the other constituents of the body the case is different; the glandular division of epithelial tissue in some parts responds promptly to nervous impulses, whilst in others no nervous impulse has been shown to have any effect. The various kinds of connective tissue, epidermic cells, the ciliated cells, and the reproductive cell do not visibly respond to any nerve impulse; and the blood corpuscles are out of the range of nervous impulses. Thus, large portions of the organism live their own lives uninfluenced, except indirectly, by the storms and stresses of the central nervous system. Even when nervous impulses can strikingly affect the activity of a tissue their action is limited, they cannot modify it in all the various ways in which it is modified by the inherent nature of the tissue and the character of the surrounding fluid. The essential effect of a nervous impulse appears to be to modify the amount of energy set free as work. The unstriated muscular tissue and glands are innervated by what is sometimes termed the "involuntary" or "autonomic" nervous system. Though these two classes of tissues differ so widely, their nervous supply has common features, and belongs to the same system. The arrangement of this involuntary system has some peculiar characters, of which the most distinctive is that the nerves do not run uninterruptedly to the periphery, they end in nerve cells, and the nerve cells send off the fibres, which run to the periphery. There is thus an intermediate station. This is in great contrast to the nerves passing to voluntary muscles, which run direct to the tissues, and have no nerve cells on their course. The nerve fibres proximal to the nerve cells are termed preganglionic, those distal postganglionic. This involuntary nervous system is divided into at least two divisions, the most extensive of which is called the sympathetic. The preganglionic fibres of the sympathetic all arise from that portion of the spinal cord which lies between the origin of the voluntary nerves of the arms and that of those of the legs. The fibres given off by the ganglia of this system, *i.e.*, postganglionic fibres, run to the involuntary tissues of all parts of the body. The second division of the involuntary nervous system forms an additional supply for certain parts of the body; it arises in the cranial and sacral regions of the cerebro-spinal axis, and supplies the muscular coats of the alimentary canal and certain structures connected developmentally with the anterior and posterior portions of this canal. They do not send any fibres to its blood-vessels. These cranial and sacral involuntary nerves differ from those of those issuing from the middle region of the spinal cord. The latter never differ in the kind of effect they produce, but only in the degree of effect; whereas the former may produce effects which are different in quality, *e.g.*, stimulation of them may lead to inhibition.

This inhibition has the characteristic feature that a certain state of activity ceases, and must be due to some direct action on the tissue of the nervous impulse. Inhibited tissues have a great tendency to contract of themselves—that is, they form very unstable substances.

If a voluntary nerve be divided, regeneration is effected by the proximal end growing down the nerve-sheath to the muscle originally innervated. Similarly, if a preganglionic nerve be divided the proximal end grows downwards—to end, as did its predecessors, in a nerve ganglion. Now the nerve fibres which end in a ganglion are rarely, if ever, all of one kind. Thus, of those which run to the superior cervical ganglion some cause the eyelids to move apart, others cause pallor of the face, and so on. Hence it would seem hopeless for the regenerating fibres as they grow again into the ganglion to find, each, the proper kind of nerve-cell. Nevertheless nearly all of them succeed in doing this. The ingrowing nerve fibres pass between and about hundreds of near relations until they find their immediate stock, for which they have seemingly a preference.

**Innervation of the Blood-Vessels of the Brain.**—The question of the vasomotor supply of the cerebral blood-vessels may at last be said to be settled definitely in the affirmative. In the early part of the century nerve-flexures were traced along the carotid and vertebral arteries. In 1894 Andriezen stated that vasomotor nerves could be stained by a modified Golgi's method, forming around the smaller pial vessels a fine non-anastomosing plexus of fibres lying between outer and muscular coats, from which terminal fibrils run to end in bulb-like enlargements against the muscle cells. A year later Gulland confirmed this, and he was supported by Morison and Obersteiner. Huber[2] has also recently by the use of methylene blue intra vitam arrived at similar results. He states that bundles of nerve fibres, both medullated and non-medullated, form a fine plexus lying on the muscular coat. From this issue fibrils which terminate in fine granule-like points abutting on the muscular cells. In addition to these vasomotor fibres other fibres are also seen which lose their medullary sheath, and terminate as free varicose fibrils in the adventitia and connective tissue of the pia; these are probably sensorial endings. The chain of evidence, therefore, in favour of the occurrence of a vasomotor supply of the pial blood-vessels is now complete, and the facts of pathology and experimental physiology must be interpreted anew in the light of these discoveries.

**The Sensory Tracts of the Central Nervous System.**—Long[3] has published an important memoir on the pathways of general sensibility. He abandons the view that there is a decussation of sensory fibres in the spinal cord. The afferent fibres of the spinal cord give forth collaterals, which terminate freely in the grey substance of the cornu on the corresponding side, while the main branches ascend and descend for varying lengths. The ascending branches terminate in the nuclei of Goll and Burdach. The afferent terminations of the cranial nerves are similarly distributed to nuclei homologous with those of Goll and Burdach. From these nuclei arises the fillet constituting the upper part of the sensory pathway. But this pathway is broken in the optic thalamus; the view of Flechsig that the fillet fibres pass uninterruptedly to the cortex is not confirmed. The median fillet terminates in the external nucleus and the centre median of the thalmus. From the thalmus various fibres proceed to different parts of the cortex. There is no special aggregation of sensorial fibres in the hindmost part of the internal capsule.

**Integrity of Anterior Cornual Cells.**—Warrington[4] has recently made experiments on the effects of cutting posterior roots. He finds in the cat that if the seventh and eighth post-thoracic posterior roots be cut, the postero-external group of cells of the anterior cornu of the same segments presents certain structural alterations—revealed by Nissl's stain. This group of cells had been previously shown to innervate the muscles of the sole of the foot—concerned in grasping movements, and is richly supplied with collaterals from the corresponding posterior roots. The section of these posterior roots causes marked loss of tone ataxia and impairment of movement. The structural alterations in the nerve cells thus form a link in the chain of evidence, and they are thus found most marked in those segments and parts of segments which especially depend on reflex impulses as an incitement to efficient discharge.

[1] Langley, Lancet, Sept. 23, 1899. [2] Huber, Jour. of Comp. Neurology, Mar., 1899. [3] Long, Thèse de Paris, 1899. [4] Warrington, Jour. of Phys., 1898 and 1899.

